# Single-Cell Analysis Using Machine Learning Techniques and Its Application to Medical Research

**DOI:** 10.3390/biomedicines9111513

**Published:** 2021-10-21

**Authors:** Ken Asada, Ken Takasawa, Hidenori Machino, Satoshi Takahashi, Norio Shinkai, Amina Bolatkan, Kazuma Kobayashi, Masaaki Komatsu, Syuzo Kaneko, Koji Okamoto, Ryuji Hamamoto

**Affiliations:** 1 Cancer Translational Research Team, RIKEN Center for Advanced Intelligence Project, 1-4-1 Nihonbashi, Chuo-ku, Tokyo 103-0027, Japan; ktakazaw@ncc.go.jp (K.T.); hmachino@ncc.go.jp (H.M.); satoshi.takahashi.fy@riken.jp (S.T.); norio.shinkai@riken.jp (N.S.); amina.bolatkan@riken.jp (A.B.); maskomat@ncc.go.jp (M.K.); 2 Department of NCC Cancer Science, Graduate School of Medical and Dental Sciences, Tokyo Medical and Dental University, 1-5-45 Yushima, Bunkyo-ku, Tokyo 113-8510, Japan; 3 Division of Medical AI Research and Development, National Cancer Center Research Institute, 5-1-1 Tsukiji, Chuo-ku, Tokyo 104-0045, Japan; kazumkob@ncc.go.jp (K.K.); sykaneko@ncc.go.jp (S.K.); 4 Division of Cancer Differentiation, National Cancer Center Research Institute, 5-1-1 Tsukiji, Chuo-ku, Tokyo 104-0045, Japan; kojokamo@ncc.go.jp

**Keywords:** single-cell analysis, next-generation sequencing, machine learning, multi-omics analysis

## Abstract

In recent years, the diversity of cancer cells in tumor tissues as a result of intratumor heterogeneity has attracted attention. In particular, the development of single-cell analysis technology has made a significant contribution to the field; technologies that are centered on single-cell RNA sequencing (scRNA-seq) have been reported to analyze cancer constituent cells, identify cell groups responsible for therapeutic resistance, and analyze gene signatures of resistant cell groups. However, although single-cell analysis is a powerful tool, various issues have been reported, including batch effects and transcriptional noise due to gene expression variation and mRNA degradation. To overcome these issues, machine learning techniques are currently being introduced for single-cell analysis, and promising results are being reported. In addition, machine learning has also been used in various ways for single-cell analysis, such as single-cell assay of transposase accessible chromatin sequencing (ATAC-seq), chromatin immunoprecipitation sequencing (ChIP-seq) analysis, and multi-omics analysis; thus, it contributes to a deeper understanding of the characteristics of human diseases, especially cancer, and supports clinical applications. In this review, we present a comprehensive introduction to the implementation of machine learning techniques in medical research for single-cell analysis, and discuss their usefulness and future potential.

## 1. Introduction

Cells are the smallest units of life, and a wide variety of them exist in living organisms. Through the developmental differentiation process, they form tissues and organs with different lineages and functions, making the particular body a living organism. In addition, it operates biological activities through mutual reactions and intercellular communication in response to signals from inside and outside the cell, as well as environmental changes. Therefore, understanding life at the single-cell level is essential for medical research to understand and control diseases, which relate to alterations of the mechanisms of life. For example, cancer tissues are composed of cancer cells and their surrounding non-cancerous cells, such as cancer-associated fibroblasts, macrophages, other blood system cells, and blood endothelial cells, and these intercellular networks play an important role in cancer survival and proliferation [1]. In particular, cancer cells are not a population of cells with uniform characteristics; genetic mutations and epigenetic modifications give rise to a population of cancer cells with different characteristics, such as cancer stem cells and differentiated cells [2,3]. Such cellular diversity in cancer tissues is the root of the contribution of malignant traits such as tumorigenicity, anticancer drug resistance, and metastatic potential of cancer cells, and it is considered to be the essential cause of refractory cancer. Therefore, analysis at a single-cell level is important because understanding cancer tissue diversity is expected to contribute to the establishment of cancer therapies.

An attempt to isolate cells from tissues and understand their properties of individual cells was first reported in 1992; the construction of an expression profile of a single living cell from the rat hippocampus was demonstrated through quantitative analysis using DNA gel blotting [4]. Subsequently, RT-PCR analysis in single cells was performed [5], and in 1998, fluorescence in situ hybridization (FISH) enabled quantitative analysis of mRNA molecules in single cells [6]. A comprehensive one-cell gene expression analysis was initially performed using microarray analysis [7]. Subsequently, a method that drastically improved the efficiency of cDNA synthesis from a single cell was reported [8]; based on that method, the first single-cell RNA-seq (scRNA-seq) method using next-generation sequencing was reported in 2009 [9]. This was the beginning of a rapid spread of research using comprehensive one-cell gene expression analysis. However, various problems have been identified as challenges in single-cell analysis. Among them, the critical issues are “sensitivity and accuracy” and “linkage with spatial information”; hence, the introduction of new technology is required.

In recent years, the expectations for artificial intelligence (AI) have increased greatly, and machine learning (ML) technology has made remarkable progress, including the rise of deep learning technology [10,11]. It is important to note that ML has been actively used in the fields of medical and biological research [12,13,14,15,16,17,18,19,20,21,22,23], and it has already been used in actual clinical practice [24,25,26]. Single-cell analysis is another field in which ML techniques have been used to overcome challenges and make more effective use of its results. Given the ability of ML technology to extract features from large-scale data, we believe that its use is essential for single-cell analysis, and it can be applied in various ways. In this review, we present a comprehensive overview of the use of ML techniques in the field of single-cell analysis and discuss their usefulness and future development.

## 2. History of Single-Cell Analysis

In 1992, gene expression analysis using a single neuron was first reported in a quantitative analysis using DNA gel blotting [4]. Subsequently, RT-PCR and FISH in single cells were reported [5,6], and in the late 1990s, microarray technology was used to analyze the expression of thousands of genes [27,28,29]. The central nervous system and neurons consist of multiple cell types; in other words, different regions correspond to different functions. Therefore, single-cell analyses have been actively investigated in this field. For example, the combination of two methodologies, i.e., laser capture microdissection for selective sample collection, and the aforementioned microarray technology for analyzing thousands of genes in a single sample, were used to disclose cellular landscapes [7,30,31,32].

Next-generation sequencing (NGS) technology is now widely used in laboratories as well as in hospitals to aid decision-making for guided precision medicine [33]. Additionally, it is necessary to use big data and multi-omics data obtained by NGS to achieve precision oncology [34]. However, NGS analysis requires hundreds to thousands of cells as a material; this creates a technical barrier as samples (or cells) are difficult to obtain. Thus, apart from the biological aspect, the first scRNA-seq analysis using the SOLiD system was reported from a realistic experimental point of view, and it was quickly and widely accepted [9]. Importantly, it was reported only a year after the first RNA-seq with bulk cell samples was published [35,36]. The scRNA-analysis introduced by Tang et al. was composed of two parts: (1) isolation of a single blastomere by mouth pipetting, and (2) cDNA synthesis from a single cell. Their method enables single-cell transcriptomic analysis that can detect 75% more gene expression than previous microarray-based methods. The basic approach as proposed by Tang et al., and especially the flow chart of cell isolation followed by NGS, remains unaltered. Yet other researchers have improved their application. For example, Islam et al. developed a custom-built semi-automated cell picker that collects a single cell into each 96-well capture plate [37], while Kivioja et al. reported the addition of unique molecular identifiers (UMIs) to cDNA during a single-cell reverse-transcription reaction to minimize PCR amplification errors [38]. In this method, a random 10-base-pair DNA tag was added to the 5′ adaptor for sequencing. Theoretically, more than 100 million (4^10^ = 1,048,576) UMIs are available and they bind to each mRNA; therefore, we can accurately estimate the amount of input mRNA by counting UMIs.

The first reported scRNA-seq analysis using a microfluidic system (Fluidigm) could analyze up to 2000 single cells at a time [39], which laid the basis for high-throughput analysis. Pico-well and nano-droplet technology lowered the cost of scRNA-seq analysis and further facilitated high-throughput analysis [40,41,42]. In 2017, the combinatorial indexing method sci-RNA-seq (GemCode), which uses gel bead-in-emulsion (GEM) from 10x Genomics, enhanced the reproducibility and accelerated the high-throughput performance [43,44]. In Table 1, we summarize the widely accepted scRNA-seq methods.

The fundamental idea for conducting a single-cell analysis is mainly to profile cellular divergence, analyze a cell population, clarify heterogeneity of microenvironment, and map the developmental landscape spatiotemporally [56,57,58,59,60,61,62,63,64,65,66]. Additionally, function-associated methodologies such as Patch-seq, a technique that combines whole-cell patch-clamp recording, immunohistochemistry, and scRNA-seq to improve classification of neural cell types as well as functional characterization [67,68,69], and in situ hybridization for single-cell analysis to reveal mRNA splicing variants have been reported [70].

## 3. Single-Cell mRNA Analysis Using Machine Learning

**Figure 1:**
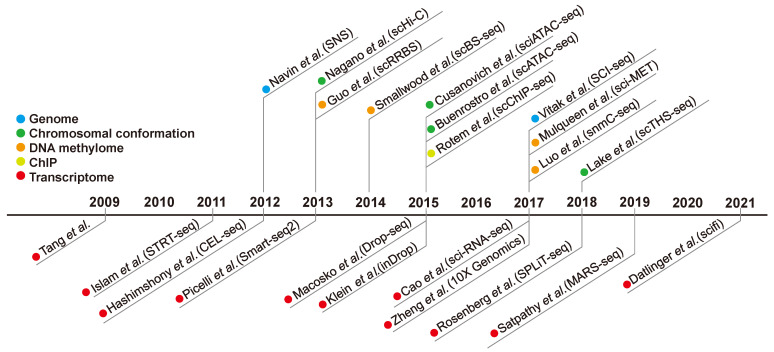
Timeline of technical progress of single-cell analysis. Each method was divided into five categories (genome, chromosomal conformation, DNA methylome, ChIP, and transcriptome) and identified by different colors.

Currently, single-cell analysis contributes significantly to not only transcriptomic analysis but also genomic analysis, such as in identifying single-nucleotide mutations in cancer [71,72,73,74] and epigenomics such as DNA methylome [75,76,77], ChIP-seq analysis and chromatin-accessibility [78,79,80] (Figure 1).

In this section, we discuss the technical aspects of scRNA-seq with ML. scRNA data can be obtained from whole cells or fractionated samples. Similar to the usual RNA-seq methods, we can analyze scRNA-seq data from whole cell samples either to classify cells of origin or cell types [81]. For fractionated samples in particular, nuclear fractionation, known as single-nucleus RNA-seq (snRNA-seq), has been reported [82,83]; moreover, snRNA-seq is applicable even in frozen human tumor tissue samples [84]. Although we do not discuss the details here, single-cell technology utilizes other omics data such as single-cell DNA sequencing (scDNA-seq), which can detect copy number variations (CNVs) as well as clonal populations [72]; additionally, it can be used for single-cell whole genome analysis [85], DNA methylation [86], or in situ sequencing known as FISSEQ [87], MERFISH [88], and seqFISH [89]. Moreover, scRNA-seq driven classification in metastatic lung adenocarcinoma shows the feasibility of revealing cellular dynamics, tumor intrinsic factors, and novel signatures that are associated with patient outcome [90].

**Figure 2:**
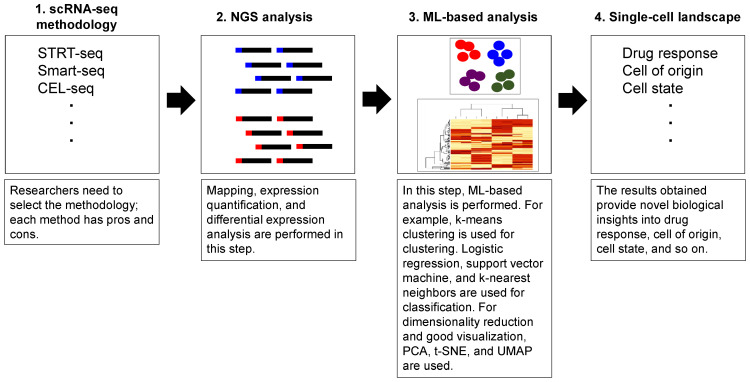
Schematic diagram of pipeline of scRNA-seq analysis. NGS data (input data or big data) is converted to actionable data using ML. Although each scRNA-seq technology has its own optimized protocol, in general, tissues or cells are first dissociated to disaggregated into single cells. At this point, high cell viability (>70%) and less than 30 μm of cell size in diameter are required from fresh samples to extract RNA. Then, using isolated RNA, cDNA is synthesized for NGS. NGS data (input data or big data) is converted to actionable data using ML such as cell type identification (classification) or subtypes identification (clustering). The obtained results (output) should be carefully reviewed if they support the research hypothesis. Besides, it is also possible to come up with new ideas with obtained scRNA-seq results.

Generally, scRNA-seq is performed with the aim of cell-type identification (classification); it is important for metastatic tumors, cell state analysis to categorize immune cells, tumor microenvironment to analyze heterogeneity, or can be performed for expression subtype analysis (clustering). To achieve the aforementioned research purpose, scRNA analysis is often performed using ML techniques. The general implementation of scRNA-seq analysis is shown in Figure 2. Although scRNA-seq is a powerful tool to address many scientific interests, technically, different batches of scRNA-seq data cause batch effects that reflect different cell populations, protocols, or technical variations. To manage these datasets for analysis, the mutual nearest neighbors (MNNs) batch-effect correction method, deep transfer learning (BERMUDA) method, and another MNN-based method named Scanorama that identifies datasets with the same cell types to cluster (not by different batches) were reported to correct batch effects [91,92,93]. Additionally, an autoencoder is a useful technique for extracting features that are associated with clinical outcomes from a high dimensional dataset [12,19,94]. The unsupervised deep embedding algorithm obtained the initial parameters from an autoencoder and learned non-linear mapping from the original scRNA-seq data space to a low-dimensional space; it showed biological interpretability through improving the clustering accuracy by removing batch effects [95]. In addition to batch effects, scRNA-seq has certain limitations such as transcriptional noises, including variability of gene expression and mRNA degradation. To overcome these problems, single-cell variational inference (scVI) based on a hierarchical Bayesian model using stochastic optimization and deep neural networks for probabilistic representation and analysis of gene expression have been reported [96]. Another autoencoder-based algorithm, i.e., single-cell decomposition using a hierarchical autoencoder (scDHA), showed the visualization of the transcriptome landscape as well as cell classification and pseudo-time inference with the combination of a non-negative kernel autoencoder to remove insignificant features; the second module of a stacked Bayesian autoencoder was used to compress data [97]. The application of a sparsely connected autoencoder was used to query cell subpopulations to investigate functional features rather than to classify cell subpopulations [98]; the single-cell graph neural network (scGNN) comprised multiple autoencoders, such as feature, graph, cluster, and imputation autoencoders; they successfully represented Alzheimer’s disease-related neural development and mechanisms [99].

From the aspect of multi-omics analysis, scRNA-seq can be combined with other NGS methods such as DNA methylation or FISH [100,101]. Seurat was initially developed to profile scRNA-seq datasets of different cell conditions (i.e., either resting or stimulated), and beyond species (either humans or mice) [102]. Later, integrated analysis with other omics data—RNA-seq, single-cell protein, and spatial analysis—was reported for a comprehensive understanding of cellular events using the advanced R tool Seurat version 3 [103]. The linked inference of genomic experimental relationships (LIGER) algorithm leveraging integrated non-negative matrix factorization (NMF) described common and uncommon features of cell identity. The use of scRNA-seq and DNA methylation data revealed the mechanisms of cell-type-specific epigenomic regulation [104]. Additionally, single-cell molecular epigenomics with ML has the potential to reveal a cutting-edge field, i.e., typically highly heterogenous cancer tissues and cancer-associated fibroblasts, which are major constituents and significantly heterogeneous to decode a tumor microenvironment [66,105].

On top of that, scRNA and chromatin accessibility (single-cell assay for transposase-accessible chromatin sequencing; scATAC-seq), have been reported to identify transcriptional and functional differences between hematopoietic stem cells from liver and bone marrow [106]. Thus, this topic is discussed in the next section.

## 4. Single-Cell ATAC-seq and ChIP-seq Analysis Using Machine Learning

During the last decade, analyses of chromatin accessibility have become essential methods to investigate the epigenetic profiles of various cell types. DNase-seq [107] and FAIRE-seq [108] are pioneering technologies for analyzing chromatin accessibility; however, the requirement of a large amount of starting material limits its wide clinical application. Subsequently, ATAC-seq [109] was introduced in 2013 and has rapidly expanded its application, taking advantage of simple protocol involving Tn5 insertion followed by PCR and less requirements of sample volume. The notable advantage is its success in single-cell analysis, which was reported by two independent research groups as “scATAC-seq” and “sciATAC-seq” in 2015 [79,80]. Furthermore, the recently developed droplet-based single-cell combinatorial indexing for ATAC-seq (dsciATAC-seq) enabled large-scale and high-throughput profiling of single-cell epigenomes [110].

Simultaneously, various analytical tools have been developed to investigate single-cell epigenomes using single-cell ATAC-seq data. Because of the high dimensionality and sparsity of the data, the computational analysis of single-cell ATAC-seq is more challenging than that of single-cell RNA-seq. To overcome this difficulty, several unsupervised ML algorithms have been proposed. First, the chromVAR utilized transcription factor (TF) motif occurrence in the open chromatin regions to project the vector of bias-corrected deviations from individual cells onto two dimensions using t-SNE. The advantage of chromVAR is that it can be used to compute the TF binding profiles associated with significant chromatin accessibility. Furthermore, chromVAR can be applied to a collection of k-mers, enabling de novo discovery of previously unannotated motifs [111]. A related approach of chromVAR is seen in BROCKMAN, where k-mer factorization is applied [112]. SCRAT uses a number of predefined features, such as TF motif, ENCODE cluster, and MSigDB gene sets [113]. By contrast, scABC relies solely on the patterns of read counts within genomic regions to cluster cells by using unsupervised k-medoids clustering [114].

Second, natural language processing techniques have been applied to cluster cells based on similarities in chromatin accessibility. A large-scale study for constructing a single-cell atlas of in vivo mouse organs by Cusanovich et al. used latent semantic analysis (LSA) to identify cell clusters [115]. Another example is cisTopic, a probabilistic framework to discover co-accessible enhancers and stable cell states, which utilizes latent Dirichlet allocation (LDA) with a collapsed Gibbs sampler to infer “cis-regulatory topics” [116].

Third, Cicero presents an ML framework to predict cis-regulatory DNA interactions using graphical lasso [117]. Cicero uses sampling and aggregation of groups of similar cells to quantify correlations between putative regulatory elements and links these regulatory elements to target genes using unsupervised ML. Interestingly, these predicted interactions are compatible with other chromatin 3D structure data such as ChIA-PET and Hi-C [117]. Cicero is potentially focused on predicting gene expression and chromatin 3D structure, as well as chromatin accessibility from single-cell ATAC-seq data [117,118].

Finally, comprehensive pipelines of single-cell ATAC-seq such as Scasat [119] and SnapATAC [120] were recently implemented; they could make single-cell ATAC-seq analysis easier to access for every researcher regardless of their specialty.

**Figure 3:**
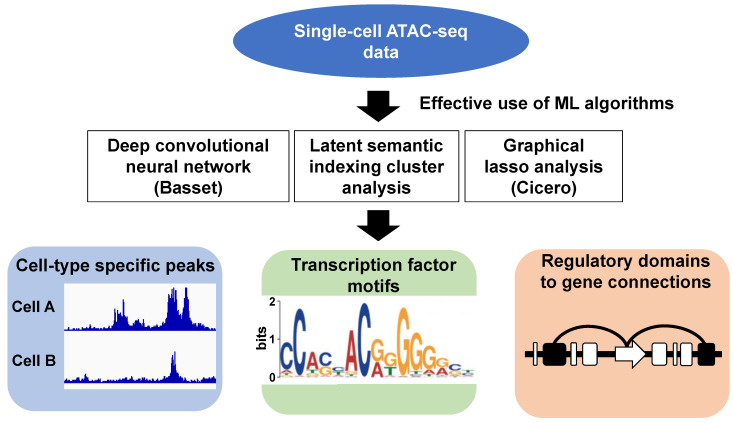
Schematic diagram of single-cell ATAC-seq analysis effectively leveraging ML. Basset is a deep convolutional neural network method to learn the functional activity of DNA sequences from genomics data. In particular, Basset allows us to simultaneously learn that cell’s chromatin accessibility code and annotate every mutation in the genome with its influence on present accessibility and latent potential for accessibility. Latent semantic indexing cluster analysis uses dimensional compression to normalize and cluster the data. Cicero uses a graphical lasso model to assign a regularized joint accessibility score, penalized by genomic distance, to each pair of “open” sites in each cluster. Hence, it is possible to find sites that were co-accessible in aggregated groups of cells within each cluster. By effectively using ML techniques in this way, we are able to predict cell clusters, transcription factors binding profiles, and cis-regulatory DNA elements (Reference [115]).

Next, we will describe some research examples of single-cell ATAC-seq using an unsupervised ML algorithm. Cusanovich et al. constructed a single-cell atlas of adult mouse tissues and characterized 85 distinct chromatin patterns across 13 different tissues [115]. After combining various ML algorithms (e.g., LSA, Cicero, and Basset) [121], they annotated key regulatory sequences and TFs in diverse mammalian cell types. Moreover, it is anticipated that this dataset can be utilized to investigate common human traits and diseases through comparing human genome with mouse genome (Figure 3).

Ranzoni et al. performed an integrative analysis of single-cell RNA-seq and ATAC-seq of human developmental hematopoiesis, revealing that there are multiple subpopulations that differ in their overall chromatin accessibility as well as lineage-specific TF activity within transcriptionally homogeneous cell clusters. They also supported the view that regulatory programming for future differentiation is primed at the chromatin level within the same cell cluster defined by scRNA-seq [106].

In conclusion, through combining single-cell ATAC-seq with other sequencing techniques and ML algorithms, it is possible to extensively define cell types and their key regulatory elements of every species, including humans. These findings provide useful resources in clinical research to investigate human disease traits and refine precision medicine.

## 5. Discussion

Recent advances in single-cell epigenomic technologies have enabled the study of genomic regulation and dynamics with unprecedented resolution. In this review, we introduced some of these innovative technologies and how they can identify the trajectory and state while taking advantage of various ML techniques. In particular, scRNA-seq is a rapidly developing technology, with several protocols published in the last few years. As previously mentioned, the most widely used methods are microwell- and droplet-based. These technologies differ in the way they tag cell-derived transcripts and how they generate libraries for sequencing. The choice of which method to use depends on the scientific question, the number of obtained cells, the depth of required information, as well as the cost. For example, droplet-based methods are best suited for characterizing tissue composition because they can handle large numbers of cells. Conversely, if only cells with known surface markers are to be analyzed, it is best to sequence a small number of cells after enrichment using flow cytometry.

Single-cell analysis allows for pseudotime analysis, which makes it possible to trace the developmental trajectory of a single cell. This indicates that single-cell epigenomic technology plays an important role in understanding cellular diversity and discovering gene regulatory mechanisms. Specifically, gene activation and cell identity are regulated by TFs, chromatin regulators, non-coding RNAs, and factors that control the chromatin 3D structure. Furthermore, the importance of cell-to-cell variability in tumor tissues is now being recognized, and single-cell epigenomic methods will be even more powerful in the future to elucidate the mechanisms of gene regulation in diverse cellular environments regarding development and disease [122].

With the growing number of single-cell epigenomic methods, a key challenge in this field is the computational combination of various single-cell “omics” methods to enable integrated cellular regulatory models. Further advanced ML is required to decode high-dimensional single-cell data [123]. In this context, previously unknown intermediate cellular states may become apparent. Indeed, several reports have evaluated intermediate states that cannot be adequately identified using known cellular markers [124,125]. Although completely reconstructing the identity of a cell may be difficult with the existing pipeline, careful construction using high-precision ML is feasible.

Furthermore, tumor immunity has been attracting attention in the field of oncology due to the recent successful clinical application of immune checkpoint inhibitors. In this context, single-cell analysis has been actively introduced to analyze the heterogeneity of immune system cells, the diversity of T and B cell repertoires, and antigen specificity. For example, to assess the breadth of T-cell activation, Want et al. profiled the TCR repertoire by single-cell sequencing when stimulated with neoantigens and identified sequence motifs that define an oligoclonal and autologous T cell response [126]. It has also been reported that accumulation of cytotoxic T cells and NK cells and increase in effector function are observed when WHSC1 is pharmacologically inhibited by single-cell analysis of immunokinetics in tumors in an experimental system using mice grafted with prostate tumor cells [127]. Importantly, the ability of ML algorithms to learn complex patterns in data is expected to be utilized in the analysis of the complex immune system. In fact, a suit of unsupervised and supervised deep learning methods, called DeepTCR, have also been proposed that can model highly complex TCR sequencing data by learning a joint representation of a TCR by CDR3 sequences and V/D/J gene usage [128]. The use of DeepTCR improved the featurization of TCRs and demonstrated its usefulness in classifying antigen-specific TCRs and extracting antigen-specific TCRs from noisy scRNA-seq and T-cell culture-based assays [128]. These results demonstrate the flexibility and capability of ML technology in analyzing the complex immune system, and further research is expected in the future.

## 6. Conclusions

As highlighted in this review, ML techniques are currently being applied to single-cell analysis in various ways, and important knowledge has been accumulated. One of the weaknesses of single-cell analysis has been the “problem of spatial information”; however, this problem is now being solved with the release of Visium, a kit for spatial transcriptome analysis from 10× Genomics, after a study using mouse brain and human breast cancer that was published by Stahl et al. in 2016 [129,130,131]. We believe that spatial transcriptome analysis is an important area in single-cell analysis where ML technology should be actively introduced in the future. In addition, ML technology plays a central role in the current development of AI. Its importance in medical AI is widely recognized, and many AI-equipped medical devices are widely used in actual clinical settings [24,132]. In fact, single-cell analysis is also expected to contribute to the realization of precision medicine by revealing gene expression profiles that lead to the prediction of patient prognosis and stratification of molecular pathologies; clinical applications of single-cell analysis are expected to progress steadily in the future through the effective use of ML technology.

## Figures and Tables

**Table 1:** scRNA-seq methodology used for analysis.

scRNA Technology	Description	Characteristics	Year	References
1. STRT (Single-cell tagged reverse transcription)-Seq	This method is based on using reverse transcriptase that possesses template-switching activity.	High accuracy of the position of 5′-end mRNA. Low cost and short time owing to the barcode strategy.	2011	[37]
2. Smart (Switching mechanism at 5′ end of RNA template)-Seq		This method enriches 5′-end mRNA and provides robust and reproducible results. SMARTer Ultra Low RNA kit for Illumina sequencing is available.	2012	[45]
3. Smart-Seq2		Improvement of reverse transcription, template switching, and pre-amplification efficacy. Exchanging one single guanylate for a locked nucleic acid (LNA) at the template-switching oligonucleotides 3′ end leads to a two-fold increase in cDNA yield.	2013	[46]
1. CEL-Seq	This method is based on in vitro transcription to reduce PCR-induced amplification bias.	This method provides highly strand specific, reproducible, linear, and sensitive results compared with PCR-based amplification and allows detection of 3′-end mRNAs using in vitro transcription. Small amounts of input RNA can be used.	2012	[47]
2. CEL-Seq2		This method is improved to achieve less amplification bias of genome sequencing, higher sensitivity, lower cost, and less working time.	2016	[48]
1. Quartz-Seq	This is a combined method of suppression PCR with poly(A) tails.	Robust suppression of byproduct synthesis with the combined techniques of poly(A) tailing with PCR amplification.	2013	[49]
2. Quartz-Seq2		The efficiency of converting initial reads into unique molecular identifiers (UMIs) has been improved because of the major improvement of poly(A) tagging, allowing for the detection of more genes.	2018	[50]
Microfluidic platform	This method is based on a microfluidic platform; single cells are captured and lysed in a microfluidic device.	The analysis of individual cells can be automated and parallelized, and cDNA can be synthesized in small-scale reactions using low-input RNA.	2014	[51]
1. Drop-Seq	This method is based on a microfluidic device that creates droplets with a single cell and reagents (such as a bead).	Digital counting of mRNA in thousands of single cells is possible.	2015	[42]
2. inDrop		A theoretical capacity to barcode tens of thousands of cells in a single run, allowing randomly labeling 3,000 cells with 99% unique labeling; many more cells can be processed by splitting a large emulsion into separate tubes.	2015	[41]
Seq-Well	This method enables the detection of a single cell in a PDMS array of more than 80,000 subnanoliter wells.	This method achieves efficient cell lysis with rapid solution exchange, while increasing the capture rate of transcripts and reducing cross-contamination by trapping biological macromolecules.	2017	[52]
Microwell-seq	This method uses microwells, which are technically simple and cost-effective through using an inexpensive device (agarose plate) for scRNA.	A simple method to profile thousands of single cells utilizing an agarose-constructed microwell array and barcoded beads to establish a convenient, simple, and cost-effective single-cell technology. This method combined existing methodologies.	2018	[53]
RamDA-seq	This method can detect a full-length total RNA expression in a single cell.	This method is highly sensitive to non-poly(A) RNAs such as lncRNAs, covers near-complete full-length transcripts, and profiles recursive splicing in >300-kb intros, detects enhancer RNAs, and their cell type-specific activity in single cells.	2018	[54]
C1 CAGE	This method is a 5′ RNA-sequencing using a C1 microfluidic system and cap analysis gene expression (CAGE) technique.	This method is an automated scRNA-seq platform using the C1 system, which can quantitatively detect the 5′ end of transcripts without bias.	2019	[55]

## Data Availability

Not applicable.
